# Capillary Bridges on Hydrophobic Surfaces: Analytical Contact Angle Determination

**DOI:** 10.1021/acs.langmuir.2c00674

**Published:** 2022-05-06

**Authors:** Norbert Nagy

**Affiliations:** Institute of Technical Physics and Materials Science, Centre for Energy Research, P.O. Box 49, H-1525 Budapest, Hungary

## Abstract

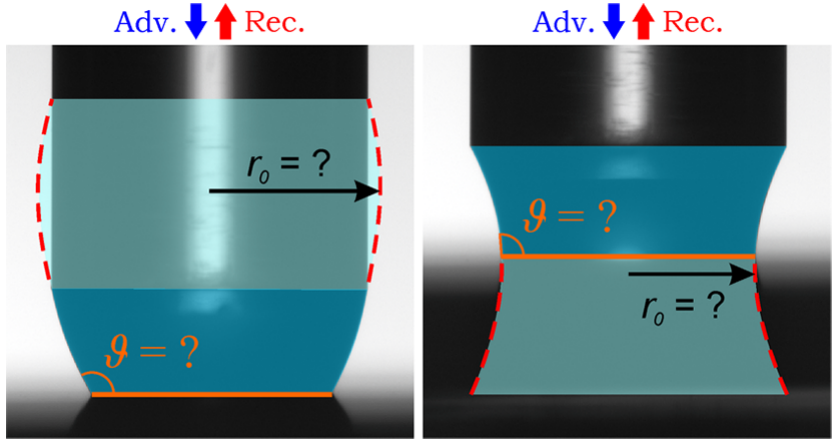

The capillary bridge probe method was introduced previously as a high-accuracy contact angle determination method relying on capillary bridges on hydrophilic and superhydrophilic surfaces [Nagy, N. Langmuir2019, 35 ( (15), ), 5202−52123091656710.1021/acs.langmuir.9b00442]. In this work, the behavior of *r*-*ϑ* type liquid bridges was studied and the contact angles were determined on hydrophobic surfaces. The equilibrium shape of these liquid bridges often does not contain the neck or haunch region. The unknown neck/haunch radius prevents analytical evaluation of the capillary bridge shape. In this work, the possible incomplete liquid bridge shapes were classified and a novel procedure was developed for the Delaunay’s analytical solution-based evaluation of these states. The parameter space of the capillary bridges was visualized and described without using dimensionless variables. As a demonstration, Cyclo Olefin Polymer and PTFE surfaces were investigated, with advancing and receding contact angles determined and compared to the results of sessile drop measurements.

## Introduction

High-accuracy contact angle determination is still an important research topic in surface science because of the central role of advancing and receding contact angles in industrial and scientific surface characterization. These quantities not only refer to the wettability of a solid surface by a liquid but also can provide information about, for example, the roughness, heterogeneity, cleanness, and energy of the solid surface, as well as about the work of solid–liquid adhesion.

The most popular contact angle measuring method is the sessile drop method because of its general usability and relative simplicity.^1^ The axisymmetric drop shape analysis has been continuously developed since its first publication.^2^ This evaluation method finds the closest solution of the Young–Laplace equation to the captured sessile drop profile; it thereby improved the precision of the sessile drop method significantly. The Wilhelmy method is considered the most accurate technique. The method is indirect: the contact angle on the sample surface is determined from the measured force acting on the sample during its immersion into the test liquid. Its major restrictions are that the length of the contact line should be known with high precision, the surface quality should be identical along the contact line, and the possible sample geometries are also limited.^3^ Recently, the capillary bridge method was developed for high-accuracy contact angle determination on spherical transparent surfaces. The spherical surface is lowered to the surface of the test liquid and the length of the evolved liquid bridge is changed after its formation. The wetted area of the spherical surface is determined from above in the function of the distance of contact line from the planar liquid surface. The evaluation is carried out based on the approximated solution of the Young–Laplace equation.^4,5^ A novel technique became popular in the last years to characterize hydrophobic surfaces: a special hydrophobic ring connected to a microelectromechanical balance holds a water drop. The investigated surface is lifted upward, and the capillary bridge is formed. The measured snap-in or spreading force corresponds to the advancing contact angle. The approach continues, and then the sample is retracted. The pull-off force is measured just before the breakage of the liquid bridge. Its value correlates to the receding contact angle.^6,7^ Furthermore, the maximum magnitude of the measured force during the retraction refers to the most stable contact angle on the investigated surface.^8^ The method was extended successfully for hydrophilic surfaces,^9^ for investigation of liquid–liquid adhesion,^10^ and for measurements of friction forces to determine sliding angles.^11^ The most recent developments and results were reviewed carefully in ref (10). A similar technique, scanning droplet adhesion microscopy, uses also the measured adhesion force of a water drop to characterize superhydrophobic surfaces^12^ because the uncertainty of the sessile drop method increases significantly for contact angles above 150°.^13^

In the last years, several research groups measured and modeled the profile of liquid bridges and the capillary force acting between parallel plates.^14,15^ Furthermore, the role of contact angle hysteresis^16,17^ and various loading rates was investigated.^18^ Liquid transfer between solid surfaces was also studied^19,20^ because of its central role in various printing processes.^21^ The capillary force was calculated, and the profile of the liquid bridge was described according to various approaches: finite element methods^14,17^ and numerical^16^ and (quasi-)analytical solutions^15,22,23^ were also applied.

The capillary bridge probe method was introduced recently. This indirect method calculates contact angles based on the measured capillary force of an *r*-*ϑ* type liquid bridge. Its capability to measure even ultralow contact angles was proved, and its high accuracy was demonstrated on hydrophilic and superhydrophilic surfaces.^24^

In this work, the behavior of *r*-*ϑ* type capillary bridges was investigated on hydrophobic surfaces. A technique is presented to overcome the difficulties of the analytical description, which emerge during these measurements: the shape of most capillary bridges does not contain the neck or haunch; that is, its radius is unknown. This procedure was demonstrated on two different hydrophobic surfaces. The complete measurement cycle could be successfully evaluated, and advancing and receding contact angles were determined. The contact angle values were compared to the results of sessile drop measurements carried out on the same surfaces. Additionally, the parameter space was visualized and depicted accurately—without using dimensionless or normalized parameters.

## Capillary Bridge Probe Method

### Principle of Measurement

**Figure 1:**
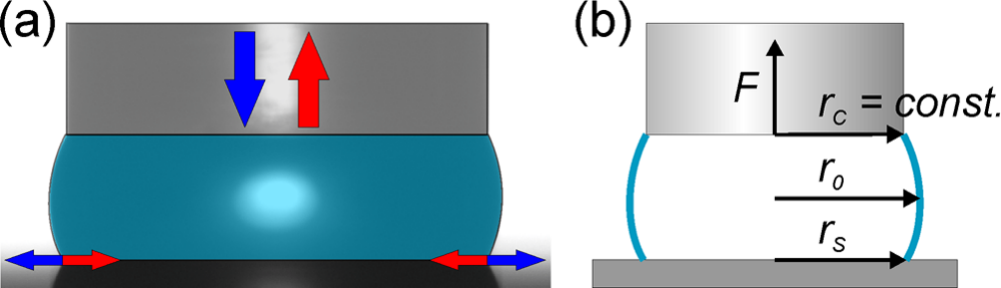
(a) Recorded image (colored) of a water capillary bridge on a PTFE surface. The blue and red arrows indicate the advancing and receding phase. (b) Schematics of an *r-ϑ* type liquid bridge with all parameters necessary for the analytical evaluation: capillary force (*F*), neck/haunch radius (*r*_0_), and surface radius (*r*_s_). The radius of the upper contact line is constant (*r*_c_ ≡ 1 mm) because of the contact line pinning on the cylinder’s rim.

The method uses a capillary bridge of the test liquid stretched between the base plane of a glass cylinder and the investigated solid surface (Figure 1a). The contact line pins at the circular edge of the cylinder; hence, the contact angle changes continuously on the upper surface (*r*-type bridge). On the sample’s surface, the determining parameter is the contact angle formed along the solid–liquid–vapor triple line (*ϑ*-type bridge).^25^ This geometry has the advantage that the advancing and receding state on the upper surface do not appear during the measurements, while it is recorded necessarily in the case of capillary bridges between different parallel plates.^16^ The liquid bridge is formed from above, from a pendant drop; therefore, the advancing contact line does not find a prewetted surface. Advancing and receding contact angles can be determined under static, quasi-static, or dynamic conditions by stepwise or continuous change of the bridge length.

### Experimental Methods

The apparatus is described in detail in ref (24). Briefly, the experimental setup is based on a classical goniometer arrangement: uniform illumination, closed sample chamber with saturated vapor of the test liquid, and imaging optics with a CMOS camera with the resolution of 1280 × 1024 pixels (spatial resolution: 3.3 μm/pixel). The glass cylinder is hooked on a force balance with the resolution of 0.1 μN, and it is mounted on a vertical actuator operated by a stepper motor with encoder resolution of 50 000 counts/rev. The diameter of the cylinder is 2 mm. In this work, the test liquid was ultrapure water (purified by a Millipore Milli-Q integral system; surface tension *γ*_water_ = 72.25 mN/m at 24 °C) with a resistivity of 18.2 MΩ·cm. The applied volumes were in the 1.6–2.5 μL range. According to the analysis of the Bond (or Eötvös) number, the gravitational effects can be neglected during the evaluation.^24,26^ All measurements were carried out in close-to-saturated water vapor (RH ≥ 85%) at 24 °C.

The measurement cycle starts with a pendant drop; hence, the volume of the test liquid can be determined with the precision of 0.01 μL. It approaches the sample surface at the chosen measuring position. After the bridge formation (snap-in), the cylinder is lowered continuously with the velocity of 0.0025 mm/s. This velocity is similar to the average speed of the stepwise movement applied in ref (24). It results in the contact line velocity remaining typically below 0.002 mm/s, and it does not exceed the value of 0.005 mm/s, even in the case of short bridges with low volumes. Therefore, the measurements are carried out in the quasi-static regime.^4,5,20^ This statement was verified by applying half of the cylinder’s velocity (0.00125 mm/s) with the same volume, and identical values were determined for capillary force and advancing and receding contact angles (see Figure S1 in the Supporting Information). The decrease of the bridge length is terminated at a chosen bridge length or contact radius. Then, the bridge length is increased (retraction phase) until the breakage of the bridge (pull-off) with the same velocity as during the approach. The image of the capillary bridge, the capillary force, and the motor’s position are recorded automatically in every fifth second.

### Evaluation

The axisymmetric capillary bridges can be described analytically, neglecting gravity. The application of the Laplace equation leads to a differential equation with boundary conditions.^15,25^ The resulting equation can be simplified by the introduction of dimensionless variables. Delaunay gave the analytic solution, which results in the Plateau’s sequence of constant mean curvature surfaces.^27^ The certain Plateau classes are determined by the dimensionless capillary pressure: *p* = *P*_c_·*r*_0_/(2γ), where *P*_c_ is the capillary pressure, *r*_0_ the neck/haunch radius, and γ the surface tension of the test liquid. According to this quantity, the capillary bridges—as constant mean curvature surfaces—are classified as nodoid with neck (*p* < 0) or haunch (*p* > 1) and unduloid with neck (0 < *p* < 0.5) or haunch (0.5 < *p* < 1). In addition, there are three special cases: catenoid (*p* = 0), cylinder (*p* = 0.5), and sphere (*p* = 1). Beside the surface tension, four known parameters are necessary for the complete analytic description (Figure 1b): the capillary force (*F*), the radius of neck or haunch (*r*_0_), and the contact radius on the lower (*r*_s_) and on the upper surface (*r*_c_). From these parameters, the length, volume, surface area, contact angles, and profile can be calculated. These equations can be found clearly tabulated in ref (28).

The capillary force (*F*) is measured by the force balance; the radius of the contact line on the upper surface is *r*_c_ ≡ 1 mm because of the contact line pinning on the cylinder’s rim. The neck/haunch radius (*r*_0_) and the surface radius (*r*_s_) are provided by the automated analysis of the captured image of the liquid bridge. The details of image analysis and more details on the evaluation can be found in ref (24). Therefore, all parameters, as well as the profile of the liquid bridge, can be determined, including the contact angle on the sample surface.

## Results and Discussion

### Complete Parameter Space

Because of the upper contact line pinning, there are only three parameters to describe all possible capillary bridges. Therefore, the properties of equilibrium nodoid and unduloid liquid bridges with *r*_c_ ≡ 1 mm were precalculated and tabulated. The relevant parameter range was discretized in 0.005 mm steps for *r*_0_ and *r*_s_, and with the resolution of 1 μN for *F*. The ranges were 0.2–1.4 mm for *r*_0_, 0.01–1.8 mm for *r*_s_, and −1100–1100 μN for *F*. This resulted in ca. 1.9 × 10^8^ lattice points, but only ca. 3.2 × 10^7^ points of them correspond to real states. The calculations were performed for water, but the resulting tables are general from the point of view of the test liquid. In the case of a different liquid with the surface tension of γ_2_, the coordinate *F* can be converted easily: *F*_2_ = γ_2_·*F*/*γ*_water_.

These parameter maps have several advantages. These tables are applied during the evaluation: the results calculated from the measured parameters are always compared to the closest tabulated equilibrium state. If the deviation of the length or the capillary force is larger than a tolerance value, it indicates that the measured bridge is not in equilibrium or it is not axisymmetric. This threshold value is 5%; that is, the measured points with higher deviation are neglected. This deviation is less than 2% for the great majority of the measurements. It is worth noting that the calculated profile is very sensitive to small deviations, resulting in conspicuous difference between the measured and the calculated silhouette. Furthermore, because of the wide open aperture of the imaging optics, the image of the capillary bridge became markedly blurred if it starts to leave the focal plane.

**Figure 2:**
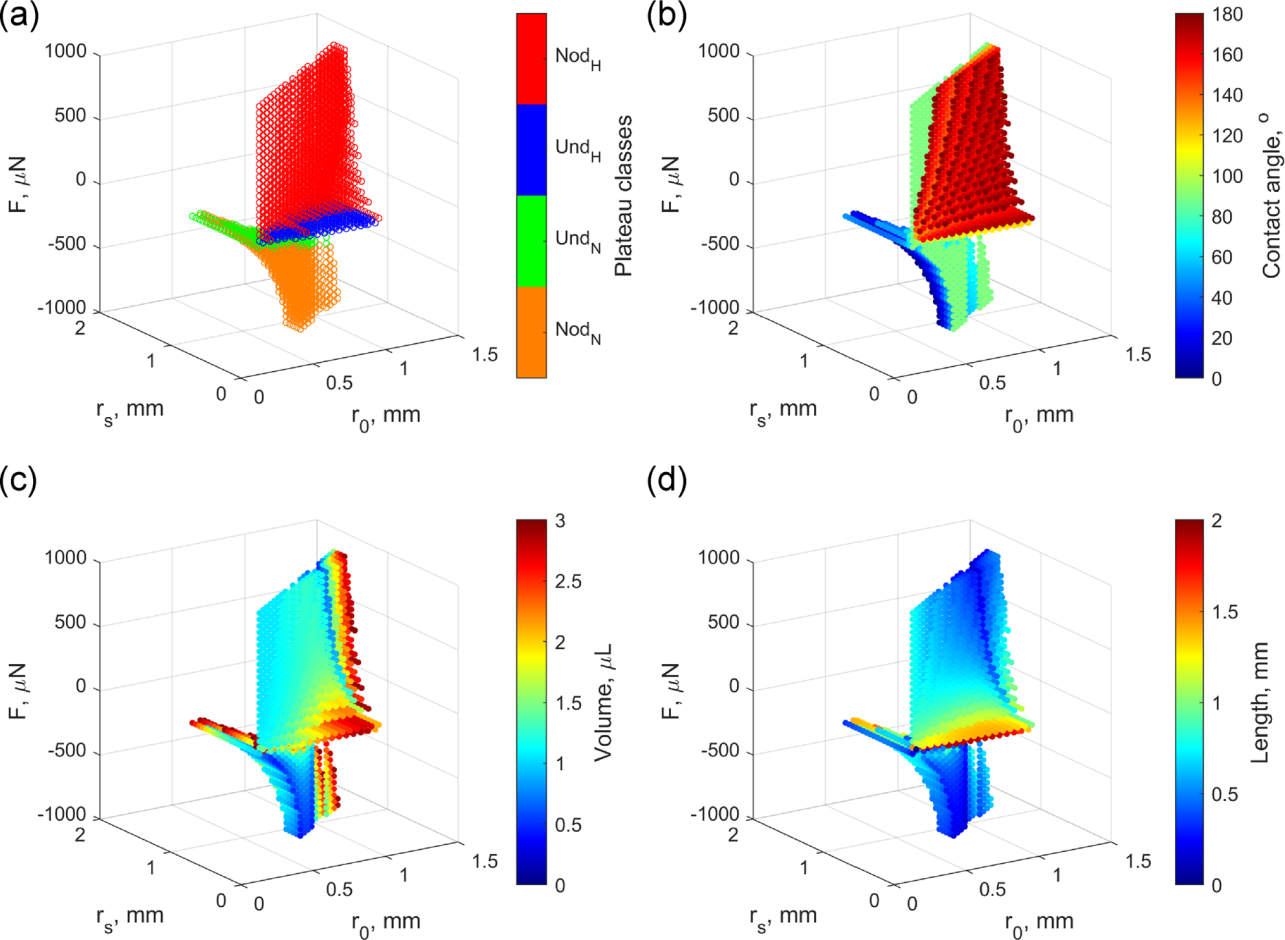
Visualization of parameter maps of possible equilibrium water capillary bridges with volumes less than 3 μL. (a) Arrangement of the major Plateau classes: nodoid with neck (Nod_N_), unduloid with neck (Und_N_), unduloid with haunch (Und_H_), and nodoid with haunch (Nod_H_). Parameter distribution of the corresponding states: (b) contact angle formed on the sample surface, (c) bridge volume, and (d) bridge length.

The precalculated look-up tables are useful also in sensitivity investigations,^24^ because the inverse problem is difficult to solve analytically.^29,30^ Furthermore, the visualization of the equilibrium states of liquid bridges in real—not in dimensionless or normalized—parameter space is expressive and informative. Figure 2 shows parameter maps of water liquid bridges with the volume less than 3 μL; the special classes (catenoid, cylinder, and sphere) are not shown for better visibility. The arrangement of the major plateau classes are plotted in Figure 2a. Nodoids with neck correspond to large magnitude of negative capillary forces, while only states of nodoid with haunch can be found in the positive force region; that is, the repulsive capillary force can be measured only for nodoids with haunch. The unduloid states with neck or haunch are located between these nodoid zones, underneath the *F* = 0 plane. This plane contains the spherical states of the liquid bridges (not plotted). In this case, the positive capillary pressure force balances the negative (attractive) contribution of the surface tension. The shape of these liquid bridges is a zone of a sphere. The corresponding contact angles on the sample surface are plotted in Figure 2b. Here, the *r*_0_ = *r*_s_ plane contains the cylindrical states (not shown) with the contact angle of 90°. This plane delimits the domains of *ϑ* < 90° and *ϑ* > 90° (but the *F* = 0 plane does not separate them strictly). The outer boundary surfaces correspond to the possible minimum and maximum *r*_s_ values with contact angles close to 180° and 0°, respectively. The states in the 0°–90° and 90°–180° range are arranged between these boundary surfaces and the *r*_0_ = *r*_s_ (*ϑ* = 90°) plane. Figure 2c,d shows the volume and the length distribution of the liquid bridges. The states of the longest (usually unduloid) bridges are located close to the *F* = 0 plane, while the large absolute capillary forces correspond to the short bridge lengths (nodoids). The states with a given bridge volume form a surface in this space with isogons of certain bridge lengths. A measured trajectory follows the isogon of advancing and receding contact angles on this surface, while it continuously changes the isogon of bridge length (see Figure S2). The maps in Figure 2 can be observed under rotation in Supporting Video S1.

### Capillary Bridge Probe Characterization of Hydrophobic Surfaces

#### Measured Bridge Properties

Capillary bridge probe measurements were carried out on hydrophobic Cyclo Olefin Polymer (henceforth Zeonex) and Polytetrafluoroethylene (henceforth PTFE) surfaces as described in Experimental Methods. The samples were prepared as follows: Cyclo Olefin Polymer (Zeonex 480R) was dissolved in toluene (1 m/V %) and spin-coated onto microscope cover slides (Menzel-Gläser) at 3000 rpm. Polytetrafluoroethylene sheets (PTFE; Kolo Ltd., Hungary) with the thickness of 1 mm were polished to remove the surface strips caused by rolling using 800–2000 grit polishing sheets. Then the PTFE samples were hot pressed between microscope glass slides (Menzel-Gläser) at ca. 210 °C for ca. 1.5 h. Finally, the surface became glossy, containing microscopic imperfections. The surface roughness was measured by atomic force microscopy on areas of 20 × 20 μm^2^. The *R*_a_ and RMS values were found to be 0.27 and 0.37 nm for the Zeonex sample and 9.1 and 11.9 nm for the PTFE surface (see Figure S3). Five measurement points were chosen on every surface, and the volumes of the capillary bridges were in the 1.6–2.5 μL range.

**Figure 3:**
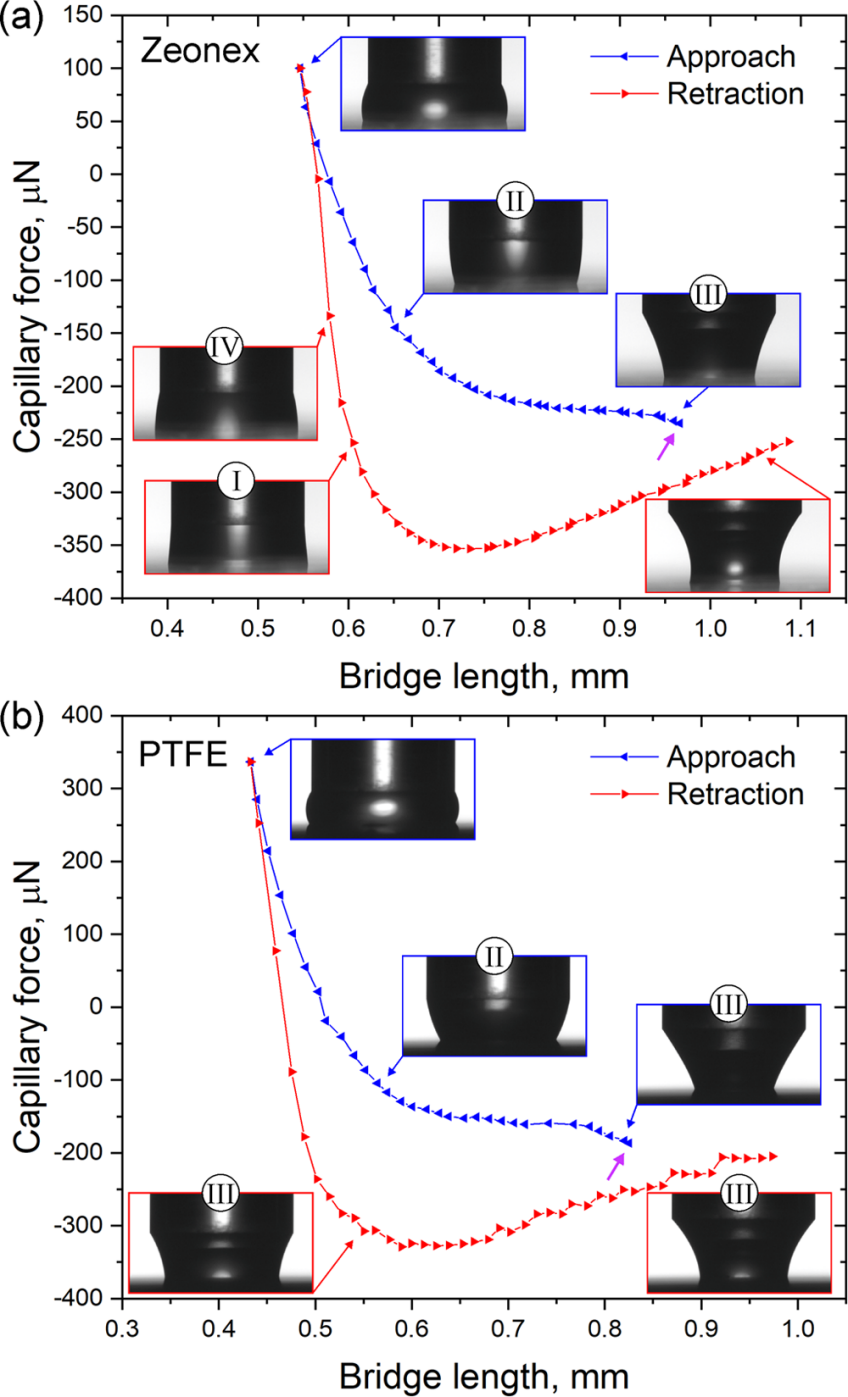
Capillary force as a function of the bridge length measured (a) on a Zeonex and (b) on a PTFE surface. The purple arrow marks the point of bridge formation. The insets show typical equilibrium states of water capillary bridges during the measurements. The Roman numerals refer to the class of the incomplete form.

Panels a and b of Figure 3 show the measured capillary force as a function of the bridge length recorded on a Zeonex and a PTFE surface, respectively. The bridge volume was 2.0 μL for Zeonex and 1.7 μL in the case of PTFE. The purple arrows mark the point of bridge formation. Both graphs show hysteresis, and they have similar character: after the snap-in, the capillary force increases with the decreasing bridge length. It changes its sign during the cycle: there is an *F* = 0 μN transition in the approaching phase and in the retraction phase, after the turn. For both surfaces, the negative (attractive) force has a minimum value in the retraction phase. Such an extrema cannot be observed on hydrophilic surfaces.^24^ After this point, the magnitude of the capillary force decreases until the pull-off. The insets show typical bridge shapes during the measurement cycle. The no-neck situation can be observed after the bridge formation in both cases. In the approaching phase, this shape transforms to a no-haunch form, and then a liquid bridge with haunch evolves. In the retraction phase, it is followed by no-haunch and no-neck states on Zeonex. Finally, a capillary bridge with neck is formed and this shape remains until the breakage of the bridge. On PTFE, no-neck shape evolves after the complete haunch states, and it does not change until the pull-off. After the breakage, a small water droplet always remains on both surfaces. The entire measurement cycle can be observed at 40× speed in Supporting Video S2 for the Zeonex surface. It can be observed that the contact line advances and recedes smoothly during the measurement; stick–slip motion can be identified just before the breakage in very stretched states. The character of the contact line motion is similar for PTFE in most cases, though stick–slip motion appears in the receding phase at a certain measuring position. Such a measurement cycle can be followed in Supporting Video S3 at 40× speed. The corresponding measured capillary force curve and the results of the evaluation show classical stick–slip character in Figure S4.

However, the majority of the measured forms do not contain the neck/haunch region. Therefore, the question is how these shapes can be evaluated analytically.

#### Evaluation of No-Neck and No-Haunch Situations: Classification of Incomplete Capillary Bridges

**Figure 4:**
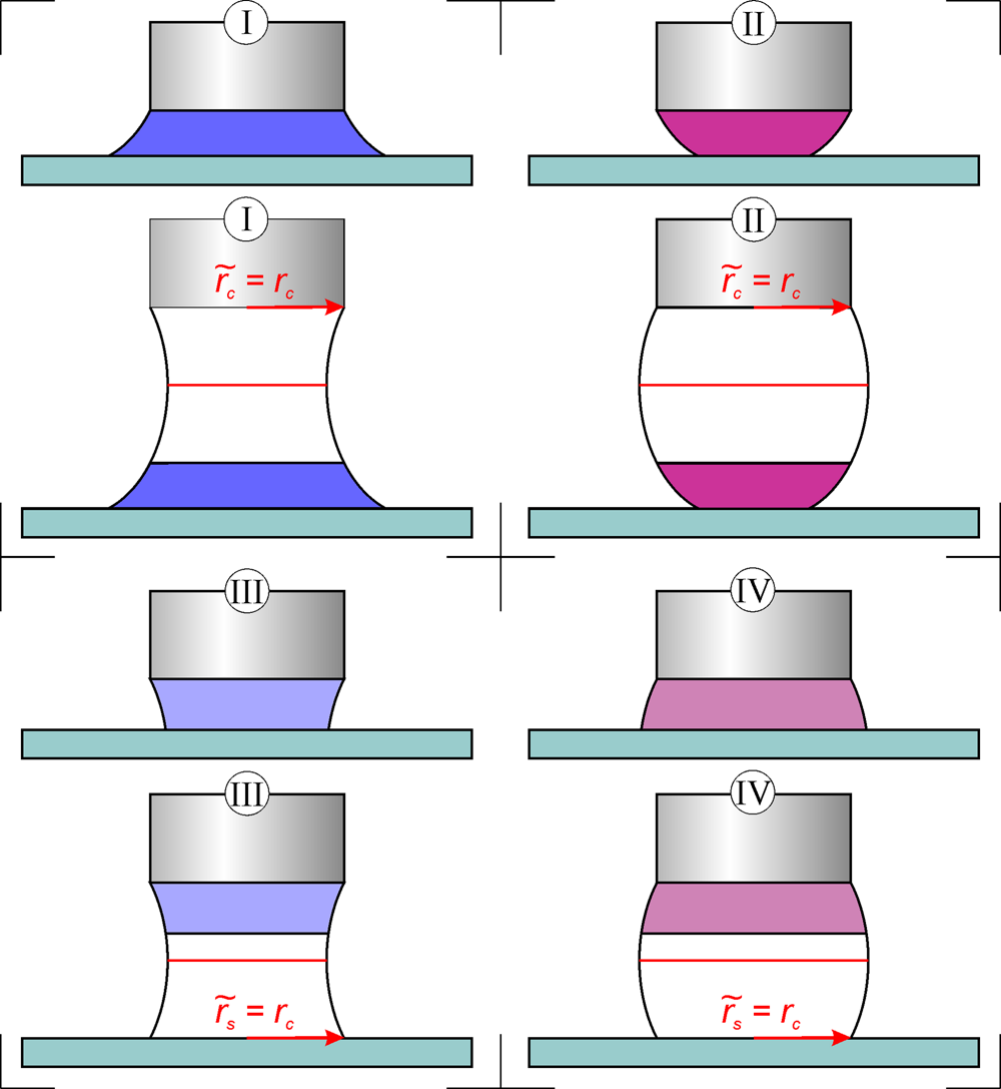
Schematics of the classification of incomplete capillary bridges: lower part of a capillary bridge with neck (I) or with haunch (II) is observable; the upper part of the bridge with neck (III) or haunch (IV) appears. The lower drawings show the idea to find identical complete bridge shapes corresponding to the different classes.

The incomplete states can be categorized into four classes (see Figure 4). The basis of the classification is that the lower part of a capillary bridge with neck (I) or with haunch (II) is observable or the upper part of a bridge with neck (III) or haunch (IV) appears. Previously, similar incomplete liquid bridges were evaluated between parallel plates analytically.^15^ In that work, the contact angle on the surface was considered as a known parameter. In our case, it is the wanted quantity. The solution is to complete these forms in an appropriate manner. This can be done because of the constant mean curvature surfaces of the capillary bridges. That is, the capillary forces are equal for every slice of a liquid bridge in the case of neglecting gravity.^15^ For class I and II, *r*_0_ is the unknown parameter to the analytic description. Hence, an identical liquid bridge should be found with the same *r*_s_ and with *r̃*_c_ ≔ *r*_c_, which has the complete form (Figure 4). This can be performed using the precalculated look-up tables. There are numerous states with the same *F* and *r*_s_ coordinates. The wanted state has different length and volume than its measured part. Therefore, the selection of the right one is not trivial. The contact angle on the surface can be estimated by polynomial fit of the profile and its derivation. This value is certainly not accurate, but the right state should be found in a ±10° range. The right one can be chosen from remaining states based on the position of the upper contact points: the profile at *r*_c_ is calculated for all remaining states, and that one is chosen which gives the closest position to the measured upper contact points. (The lower contact points should agree with the profile with the deviation of ≤0.005 mm because *r*_s_ is a measured input parameter.) Now, the identical equilibrium state was found with its missing *r*_0_ value. Therefore, the measured part can be described analytically; that is, all parameters can be computed. The area and the volume of the measured capillary bridge can be calculated by subtracting the upper part (integral from *r*_0_ to *r̃*_c_ = *r*_c_) from the lower part (integral from *r*_0_ to *r*_s_). Note that most calculations are carried out using dimensionless variables, according to the equations of ref (28).

The case of class III and IV seems to be more complicated because there are two unknown parameters: *r*_s_ and *r*_0_. Here, the solution is to look for states with symmetry to the *r*_0_ plane, i.e., with *r̃*_s_ ≔ *r*_c_ (see Figure 4). The process is similar to the case of class I and II from this point. The number of possible states with the given *F* and *r̃*_s_ ≔ *r*_c_ is decreased by the estimated value of the contact angle on the cylinder, because its value is equal to the contact angle on the lower virtual surface because of the plane symmetry. Finally, that one state is chosen which has the profile closest to the lower contact points. Thereby, the value of *r*_0_ is already known and all parameters can be calculated analytically. In this case, the bottom of the upper part (integral from *r*_0_ to *r*_s_^measured^) is subtracted from the half of the bridge (integral from *r*_0_ to *r*_c_) to calculate the volume and the area.

Making a distinction between the four different classes can be easily automated. Simple function analysis of the polynomials fitted on the bridge profile provides sufficient information to classify the captured liquid bridge. The sign of the first derivative and the position of the end points uniquely refer to the class to which the certain bridge can be assigned.

#### Contact Angle Determination

**Figure 5:**
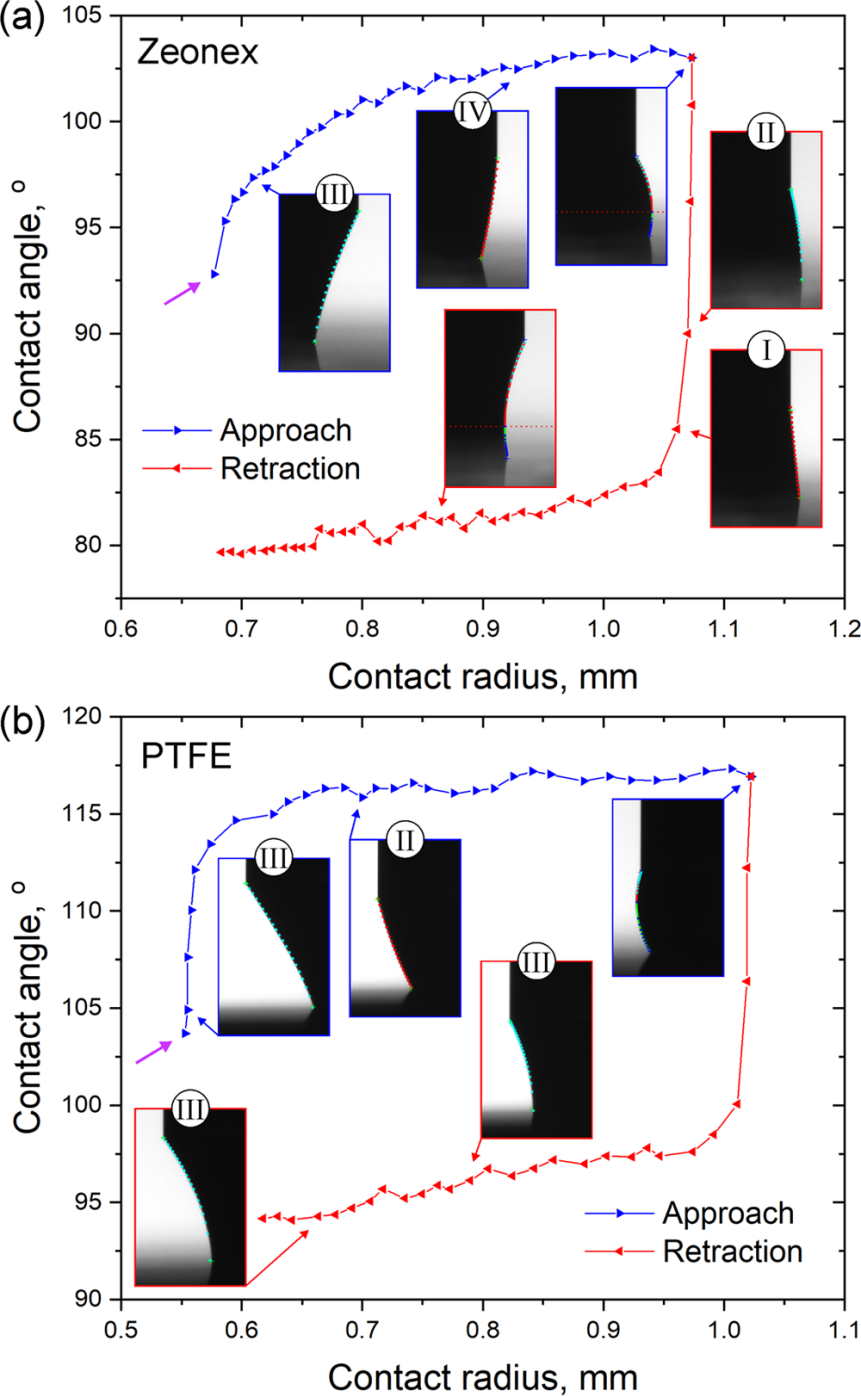
Contact angle as a function of the contact radius determined (a) on a Zeonex and (b) on a PTFE surface. The purple arrows mark the point of bridge formation. The insets show the evaluated profiles of the capillary bridges. The numbers refer to the class of the incomplete form.

Using the process described above, the whole measurement cycle can be evaluated on a hydrophobic surface. The determined contact angle as a function of the contact radius (*r*_s_) is plotted in panels a and b of Figure 5 for Zeonex and PTFE, respectively. Plotting contact angles versus contact radius is a useful representation because the change of the radius indicates that the angle actually corresponds to advancing or receding state of the contact line. The contact angles in Figure 5 were calculated based on the measured data of Figure 3. The insets show characteristic evaluated profiles for different classes. It can be seen that the captured profiles are in remarkable agreement with the calculated ones. Contact angle hysteresis can be observed on both curves during the approach–retraction cycle. After the formation of the liquid bridge, the contact angle increases to its advancing value, and this value remains stable to the end of the approaching phase. It results in at least 11–16 advancing values in every measuring position. The contact line does not start to recede immediately after the turn. When it starts to move, the receding contact angle decreases slightly during the retraction, similar to the results of evaporative drop measurements^31−34^ or to the experiences on hydrophilic surfaces.^24^ The value of the first receding contact angle is that point where the contact radius decreases significantly. For comparison, the final receding contact angle is chosen at that contact radius value where the advancing contact angle became stationary.

It is worth noting that the difference between the measured and calculated bridge length reaches and exceeds the tolerance of 5% before the pull-off. This is the reason why the last measured points were not plotted in Figure 5a,b. This difference cannot be explained by gravitational effect correlated to the increased bridge length according to the analysis of the Bond number.^20,24,26^ It seems that these highly stretched states are not in equilibrium. Neither the bridge lengths calculated from measured data (for Zeonex) nor the lengths derived from the precalculated tables (for Zeonex and PTFE) are in agreement with the measured values. In the case of large bridge volumes (∼2.5 μL), the situation is similar just after the bridge formation in the first 1–2 measured points.

The measured trajectories containing incomplete capillary bridges cannot be represented in the parameter space as was depicted in Figure S2. As an example, it can be seen in Figure S5 that the capillary force measured on a Zeonex surface is continuous in the *F*–*r*_s_ plane, but the 3D trajectory has discontinuity in *r*_0_ due to the neck-haunch transition in the advancing phase and at the haunch-neck transition during the retraction.

#### Comparison with Sessile Drop Measurements

Sessile drop measurements were carried out on the same Zeonex and PTFE surfaces for comparison. The advancing and receding contact angles were determined by applying the drop build-up technique^1,35^ in close-to-saturated vapor (RH > 85%) at 24 °C at five different measuring positions. Ultrapure water was used as test liquid. Images of the sessile drops were captured using the same apparatus. The volume of the drops was increased and decreased in 2 μL steps in the range of 6–20 μL using a Hamilton syringe. The needle of the syringe was approached and removed from the droplet with the velocity of 0.125 mm/s. The captured images were evaluated using the Young–Laplace, the ellipse fitting, and the circumcircle and difference fitting^36^ methods. It is worth noting that the difference between the contact angle values resulting by different methods remained below 0.3° for the great majority of the measurements, and typically it did not exceed 0.6°. The largest difference was found on PTFE between the Young–Laplace and elliptic fit (0.9°). However, the differently evaluated results gave the same mean and standard deviation (rounded to the nearest tenths).

**Figure 6:**
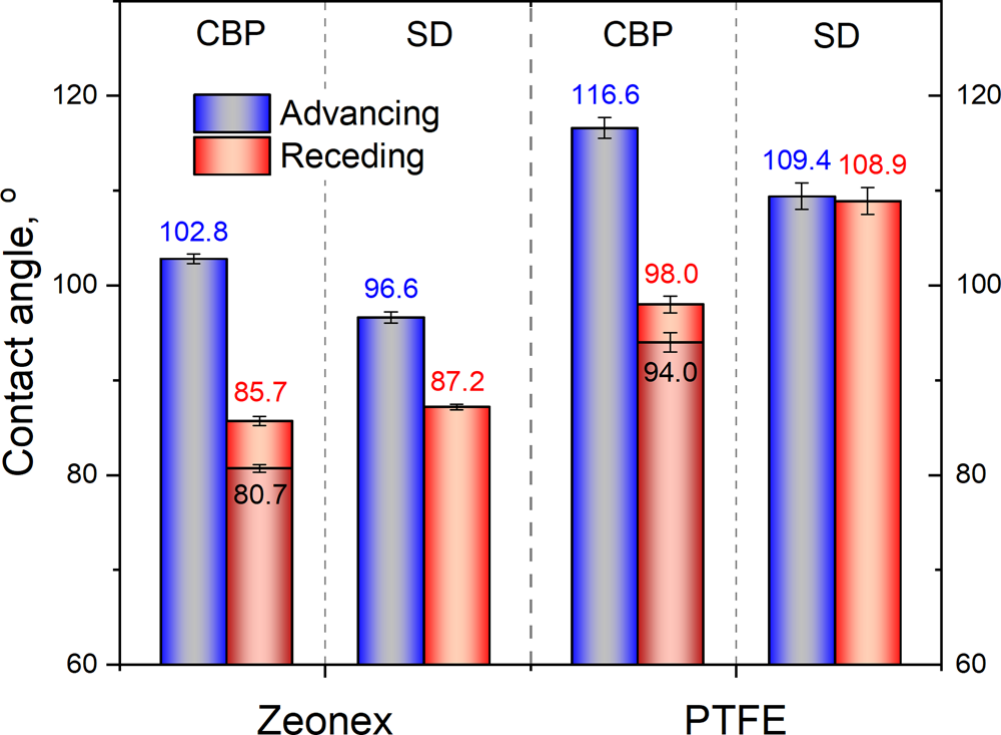
Advancing and receding contact angles determined by the capillary bridge probe (CBP) and the sessile drop (SD) methods on Zeonex and PTFE surfaces.

The comparison chart is shown in Figure 6. The tabulated results can be found in Table S1. The averages and standard deviations were calculated from the values measured at five different positions. The difference between values resulting from different measuring methods is of larger magnitude than their standard deviation. This means that the repeatability of each method is better than the comparability of the values determined by different methods, as was already stated for sessile drop, tilted plate, evaporative drop, and capillary bridge probe methods in ref (24). The provided values are plausible for both surfaces. However, a characteristic difference can be identified in the case of the PTFE surface: the results of the sessile drop method practically does not show any contact angle hysteresis, while the magnitude of the standard deviation refers to the presence of surface imperfections. However, according to the capillary bridge probe method, the hysteresis is considerable (>18°). The shorter length of the contact line can explain the higher sensitivity of this method to surface imperfections. Additionally, as a result of the high positive capillary pressure at the end of the approaching phase, wetting transition from the Cassie–Baxter to the Wenzel state may occur in the case of the PTFE sample. That is, the microscopic imperfections (Figure S3) may become completely wetted, while in the case of sessile drops some trapped air can remain in deeper pits and grooves, if the pressure does not reach the critical value for wetting transition.^37^

## Conclusions

The capillary bridge probe method was introduced and validated previously for contact angle determination on hydrophilic and superhydrophilic surfaces. The method combines the accuracy of the Wilhelmy method and the general usability of the sessile drop method. Its capability was proved to measure even ultralow contact angles (<1°) with high accuracy.^24^ In this work, the behavior of capillary bridges was studied on hydrophobic surfaces. It was shown that the majority of the equilibrium states of these *r-ϑ* type liquid bridges do not contain the neck/haunch region; therefore, the analytical description of these states is problematic. The incomplete shapes were classified, and a novel technique based on this classification and on the use of simple look-up tables was presented to overcome this difficulty. The developed procedure was successfully applied: whole measurement cycles were evaluated analytically, and the calculated profiles of the capillary bridges showed remarkable agreement with the captured silhouettes. Additionally, the complete parameter space was visualized and depicted accurately, without the use of dimensionless or normalized parameters. Advancing and receding contact angles were determined on hydrophobic Cyclo Olefin Polymer (Zeonex) and PTFE surfaces. These contact angles were compared to the results of sessile drop measurements. Both methods showed high reproducibility in the investigated 80°–120° contact angle range, and the results were found to be plausible. However, a difference could be noticed in the case of the PTFE surface: in contrast to the capillary bridge probe method, the sessile drop method did not show contact angle hysteresis on this surface. Hence, the capillary bridge probe method shows higher sensitivity to the surface imperfections.

Thereby, the capability of the capillary bridge probe method was extended to characterize also hydrophobic surfaces with high sensitivity and good repeatability. In addition, the measuring setup has the advantage that an additional force balance can easily complement existing contact angle goniometers.

## Supporting Information Available

Materials; methods; results of atomic force microscopy; results of capillary bridge probe measurements with different loading rates; measured trajectory plotted on the parameter map of capillary bridges with a certain volume; results of capillary bridge probe measurements on PTFE with stick–slip motion; discontinuities in the parameter space for incomplete states; comparison table of contact angles measured by different methods (PDF)
Supporting Video S1: rotating parameter maps (AVI)
Supporting Video S2: measurement cycle on Zeonex at 40× speed (AVI)
Supporting Video S3: measurement on PTFE with stick–slip motion at 40× speed (AVI)

## Supplementary Material

la2c00674_si_001.pdf

la2c00674_si_002.avi

la2c00674_si_003.avi

la2c00674_si_004.avi
