# Supplementary material for: Capillary Bridges on Hydrophobic Surfaces: Analytical Contact Angle Determination
Source: Langmuir. 2022 May 6;38(19):6201–8. doi: 10.1021/acs.langmuir.2c00674 (PMC9118539; doi:10.1021/acs.langmuir.2c00674)
Supplement: Supplementary file 1 — la2c00674_si_001.pdf [file la2c00674_si_001.pdf]

Supporting Information

Capillary bridges on hydrophobic surfaces: Analytical contact angle determination

*Norbert Nagy**

Institute of Technical Physics and Materials Science, Centre for Energy Research
P.O. Box 49, H-1525 Budapest, Hungary

*E-mail: nagyn@mfa.kfki.hu

Table of contents

1. Materials S2
   1.1 Test liquid S2
2. Methods S2
   2.1 Atomic Force Microscopy S2
3. Additional data S2

1. Materials

1.1 Test liquid

Ultrapure water purified by a Millipore Milli-Q integral system; resistivity: 18.2 MΩ·cm; surface tension γ = 72.25 mN/m at 24 °C measured by Wilhelmy balance method (KSV 2000) and pendant drop method (Krüss DSA 30).

2. Methods

2.1 Atomic Force Microscopy

The surface morphology and roughness of the Zeonex and PTFE samples were characterized using an AIST-NT SmartSPM 1000 atomic force microscope operated in tapping mode. The measurements were performed over 20 × 20 μm^2 areas. The measured data were analyzed using the Gwyddion software.

3. Additional data

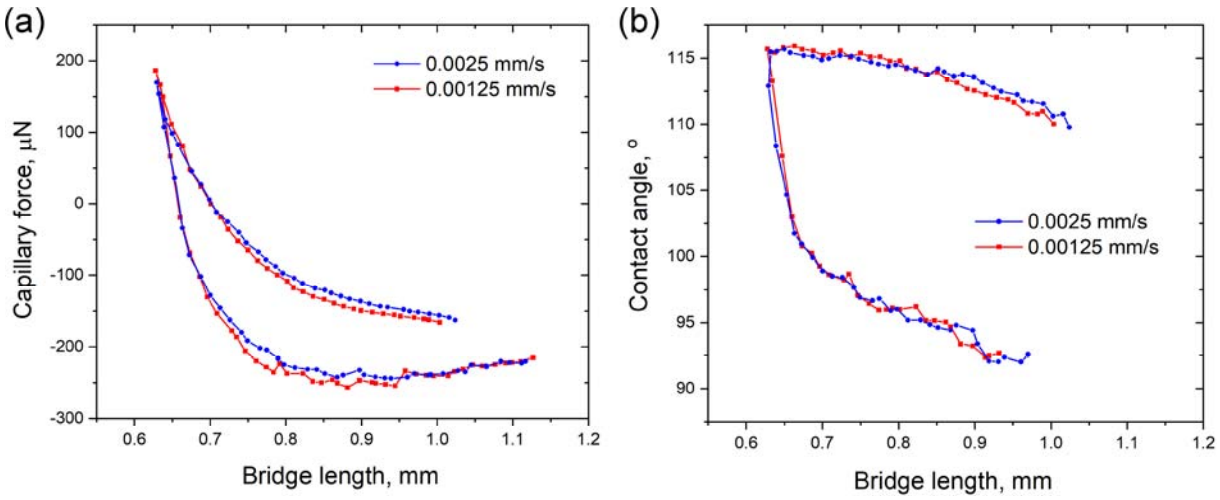

Figure S1. (a) Capillary force as a function of the capillary bridge length measured on a Zeonex sample at two different cylinder velocity. **(b)** Corresponding determined contact angles as a function of the bridge length. The measured force and the calculated contact angle values are practically equal for both velocities. The volume of the water capillary bridge was 2.5 μL.

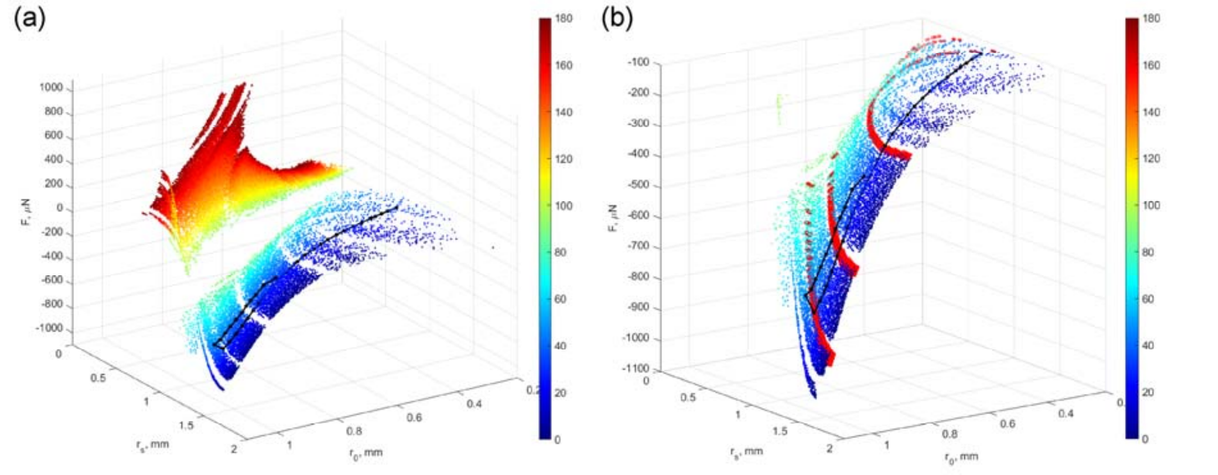

Figure S2. The black line represents a measured trajectory in the complete parameter space **(a)** and in the parameter map of hydrophilic states **(b)**. The plotted surfaces contain the states of equilibrium capillary bridges with the volume of 1.3 μL in the bridge length range of 0.4–1.1 mm. The color bar refers to the contact angle of the certain states. It can be observed that the density of the corresponding states are not uniform. The red strips in **(b)** show isogons of equal bridge lengths (lower is shorter). During the decrease of the bridge length, the trajectory follows the isogon of the advancing contact angle, and then it proceeds along the isogon of the receding contact angle in the retraction phase until the pull-off on the top of the plotted surface.

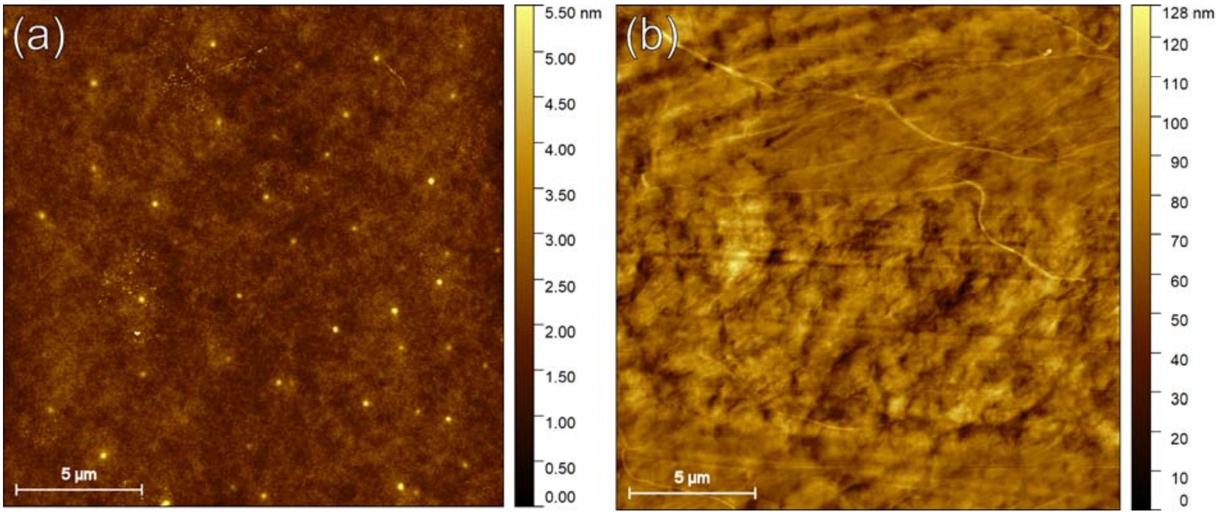

Figure S3. Height images measured by an atomic force microscope on 20 × 20 μm^2 areas of the **(a)** Zeonex sample and of the **(b)** PTFE surface. It can be observed in (b) that there is a granular region between two relatively smooth plateaus above and below.

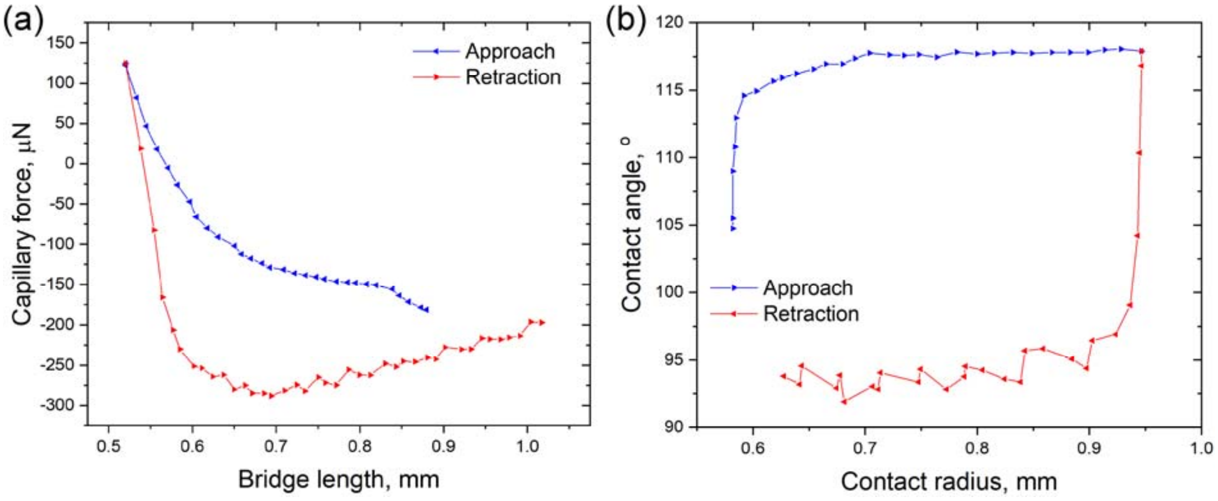

Figure 4. (a) Capillary force as a function of the bridge length measured on a PTFE surface. **(b)** Corresponding determined contact angles as a function of the contact radius. The stick-slip motion of the contact line is obvious in the receding phase. The bridge volume was 1.8 μL.

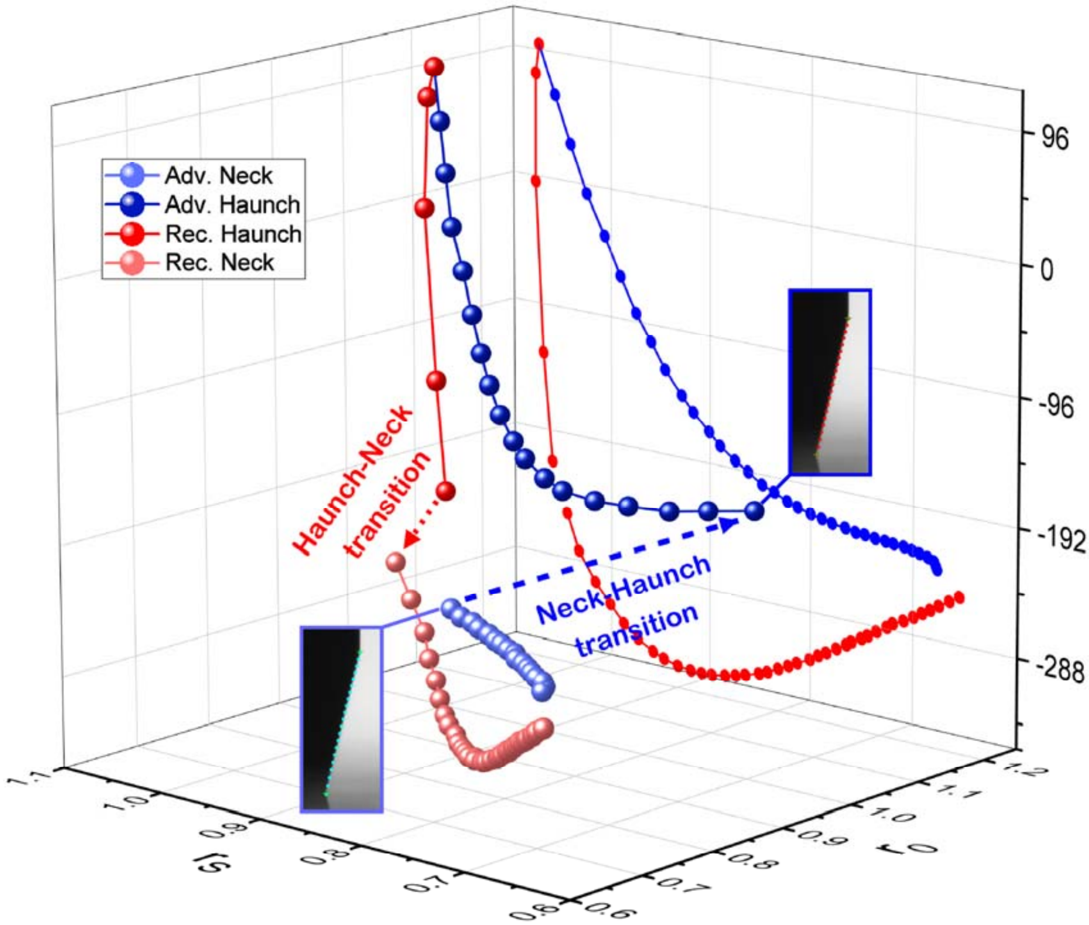

Figure S5. The capillary force measured on a Zeonex surface is continuous in the F–r_s plane, but the 3D trajectory has discontinuity in r_0 due to the neck-haunch transition in the advancing phase and at the haunch-neck transition during the retraction. The volume of the liquid bridge was 2.0 μL.

Table S1. Advancing and receding contact angles determined by the sessile droplet (SD) and capillary bridge probe (CPB) methods on the Zeonex and PTFE surfaces. The averages and standard deviations were calculated from the values measured in five different positions.

	CBP		SD	
	Advancing	Receding	Advancing	Receding
Zeonex	102.8 ± 0.5	87.7 ± 0.5 ⇒ 80.7 ± 0.4	96.6 ± 0.6	87.2 ± 0.3
PTFE	116.6 ± 1.1	98.0 ± 0.9 ⇒ 94.0 ± 1	109.4 ± 1.4	108.9 ± 1.4
